# The impact of age and electrode position on amplitude-integrated EEGs in children from 1 month to 17 years of age

**DOI:** 10.3389/fneur.2022.952193

**Published:** 2022-08-25

**Authors:** Sandra Greve, Verena Tamara Löffelhardt, Adela Della Marina, Ursula Felderhoff-Müser, Christian Dohna-Schwake, Nora Bruns

**Affiliations:** ^1^Department of Pediatrics I, Neonatology, Pediatric Intensive Care Medicine, and Pediatric Neurology, University Hospital Essen, University of Duisburg-Essen, Essen, Germany; ^2^C-TNBS, Centre for Translational Neuro- and Behavioral Sciences, University Hospital Essen, University of Duisburg-Essen, Essen, Germany

**Keywords:** amplitude-integrated EEG, neuromonitoring, pediatric intensive care, electrode position, channel, percentiles, normal values, pediatric aEEG

## Abstract

**Aim:**

Amplitude-integrated electroencephalography (aEEG) is used to monitor electrocortical activity in critically ill children but age-specific reference values are lacking. We aimed to assess the impact of age and electrode position on aEEG amplitudes and derive normal values for pediatric aEEGs from neurologically healthy children.

**Methods:**

Normal EEGs from awake children aged 1 month to 17 years (213 female, 237 male) without neurological disease or neuroactive medication were retrospectively converted into aEEGs. Two observers manually measured the upper and lower amplitude borders of the C3 – P3, C4 – P4, C3 – C4, P3 – P4, and Fp1 – Fp2 channels of the 10–20 system. Percentiles (10th, 25th, 50th, 75th, 90th) were calculated for each age group (<1 year, 1 year, 2–5 years, 6–9 years, 10–13 years, 14–17 years).

**Results:**

Amplitude heights and curves differed between channels without sex-specific differences. During the first 2 years of life, upper and lower amplitudes of all but the Fp1–Fp2 channel increased and then declined until 17 years. The decline of the upper Fp1–Fp2 amplitude began at 4 years, while the lower amplitude declined from the 1st year of life.

**Conclusions:**

aEEG interpretation must account for age and electrode positions but not for sex in infants and children.

## Introduction

In recent years, the use of amplitude-integrated EEG (aEEG) has expanded from neonatology into pediatric intensive care, driven by the need for continuous neurophysiological monitoring and the advantages of aEEG as an affordable, broadly available, and easy-to-interpret bedside technique (1). Continuous full channel EEG, the gold standard to monitor electrocortical acitivity in critically ill children, is a scarce resource with high barriers for implementation (2). As an alternative, aEEG has proven useful for the assessment of seizures and guiding antiepileptic treatment in critically ill children, making it the preferred tool by pediatric intensive care givers (1, 3–7). There is incipient but growing evidence that physiological and pathological conditions induce changes in the aEEG background pattern, e.g., sleep states, sedation, cardiac arrest, central nervous system infections, and inflammation (4, 5, 8–13). Normalization of background patterns according to a neonatal classification (14) is associated with outcome in neonates, children, and adults after hypoxic events (9, 15–17).

Neonatal aEEG classifications found that the physiological aEEG changes with gestational and postnatal age (18–21). Despite these findings and increasing aEEG use in older infants and children, reference values have not been defined for infants and children above 3.5 months of age. Specific diseases and interventions in pediatric intensive care can require adaptations to the standard electrode positions used in neonatology, making the establishment of reference values more complex than in neonates.

To address the growing need for reference values (22), we calculated aEEGs from normal EEGs recorded in awake children without cerebral disease who were not critically ill and did not receive sedatives, antiepileptic drugs or any other type of neuroactive medication. The aim was to assess whether age and electrode positions affect aEEG amplitudes and provide normal values for bedside assessment of aEEG in the PICU. We measured the upper and lower margins of five aEEG channels that are used in pediatric intensive care and calculated age-specific percentiles.

## Methods

EEGs without pathologies from awake children between 1 month and 17.9 years of age and without central nervous system disease or neuroactive medication were eligible for the study. EEGs were classified into 1-year age groups during the selection process. To avoid bias from repeated recordings in the same patient, only one EEG per one-year age group was selected from each patient.

### Ethics approval

The study was approved by the local ethics committee of the Medical Faculty of the University of Duisburg-Essen (20-9444-BO). Informed consent was not necessary according to local legislation because retrospective anonymized data were used.

### Indication for EEG recording and verification of eligibility

Most EEGs were conducted as part of the clinical routine in several conditions before the initiation of therapy, but never in critically ill children (Table 1). Our center has a large pediatric oncology department. All children undergoing chemotherapy receive an EEG before therapy in order to have a baseline finding in case of neurological complications. Another specialty of our center are solid organ transplants and bone marrow transplants (oncological and non-oncological). The same pre-therapy diagnostics are applied to these patients. Some other indications were diagnostic work-ups in suspected neurologic disease. No neurologic disease was allowed to be diagnosed at any timepoint in the patient history before or after the EEG recording until inclusion into the study. For acquired brain injury, only recordings before the insult were eligible. In oncological patients, affection of the CNS was ruled out by imaging and/or lumbal puncture as appropriate according to the underlying malignancy.

**Table 1:** Eligibility criteria.

**Inclusion criteria (all must be met)**	**Exclusion criteria (one is sufficient)**
•Age <18 years	•Acquired or inborn cerebral disease or damage
•EEG normal according routine assessment	•Antiepileptic drug use or other neuroactive medication
•Awake throughout recording	•Former preterm infant before 24 months corrected age
	•Fallen asleep during recording
	•Developmental delay
	•Photostimulation during recording^*^

In case of more than one recording within the same year of age: only one random EEG used to avoid bias from repeated measurements in the same subject.

^*^Hyperventilation was not an exclusion criterion because all EEGs were annotated. Amplitudes were only assessed until the beginning of hyperventilation.

Patient histories were checked for neuroactive substances and patients excluded if they received any type of neuroactive medication at the time of recording.

### EEG recording

Full-channel EEG was applied according to the international 10–20 system after skin preparation with OneStep EEG Gel Abrasiv plus®. An impedance check was performed, and skin preparation was repeated until impedances <5 kΩ were achieved for all electrodes, according to the requirements for standard EEG recording by the German Society for Clinical Neurophysiology and Functional Imaging. All EEGs were recorded using Neurofax EEG devices and polaris.one software v4.0.4.0 (Nihon Kohden, Tokyo, Japan).

### EEG interpretation

The EEG reader was a board-certified pediatric neurologist (ADM) with additional certificates in EEG and epileptology by the German Society for Epileptology (DGfE). Because all EEGs were interpreted by ADM or, on rare occasions, by a substitute who holds the same qualifications, no additional independent read was performed before inclusion of EEGs into the study.

### EEG conversion

**Figure 1:**
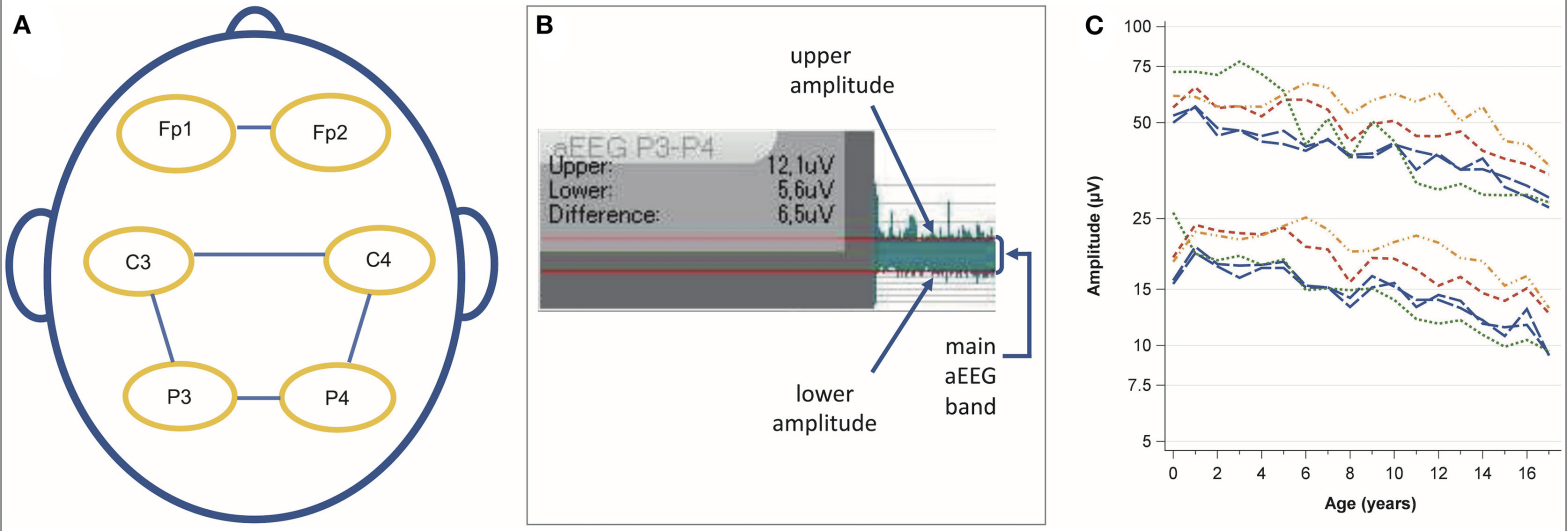
**(A)** Analyzed channels according to the 10 – 20 system. **(B)** Amplitude assessment using the integrated ruler for amplitude measurement. The red lines were manually aligned with the main aEEG band. The small spikes above and below the main band were not considered. **(C)** Evolution of the median upper and lower amplitude values with age. Blue long-dashed line: C3–P3 and C4–P4, red medium dashed line: C3–C4, green short-dashed line: Fp1–Fp2, orange dashed-dotted line: P3–P4.

aEEGs were calculated using Polaris EEG software (Polaris Trend Software QP-160AK, Nihon Kohden, Tokyo, Japan). Channels C3–C4, P3–P4, C3–P3, C4–P4, and Fp1–Fp2 of the 10–20 system were converted (Figure 1A). All channels except for the Fp1–Fp2 channel are standard for aEEG conduction in neonates. The Fp1–Fp2 channel was additionally included because in patients with head injury or after neurosurgery, the frontal positions can be the only available locations for electrode placement.

### aEEG analysis

SG and NB measured the upper and lower amplitude borders manually with the integrated tool for amplitude measurement as previously described (13) (Figure 1B).

An image of the measured tracing was saved as a PDF file for documentation purposes and quality control. The measured values were transferred manually into an Excel spreadsheet.

### Data analysis

Categorical variables are summarized as counts and relative frequencies. aEEG amplitudes are presented as age-specific percentiles (10th, 25th, 50th, 75th, and 90th). Other continuous variables are presented as means and 95% confidence intervals (CI) or standard deviation (SD) if normally distributed and as median and interquartile range (IQR) if non-normally distributed.

#### Calculation of amplitudes

For each aEEG channel, we calculated the mean of the values measured by the two raters for the upper and lower amplitudes.

#### Definition of age groups

The raw data were visualized to assess the height and evolution of amplitudes with age (Figure 1C). Next, we defined age groups to calculate percentiles (<1 year, 1 year, 2–5 years, 6–9 years, 1 −13 years, and 14–17 years). The aim was to depict the rapid amplitude changes during the first years of life, provide sufficient detail on amplitude differences at older ages, and avoid an excessive number of subgroups. The C3–P3 and C4–P4 channels were collapsed for percentile calculations because they represent the contralateral positions during 2-channel recordings. To assess sex-specific differences, we calculated means and 95 % CIs for males and females for each channel and age group.

#### Interrater reliability

Bland–Altman plots (23) were created for the upper and lower amplitudes of each channel for each year of age to rule out systematic differences in measurements between the two raters. Mean differences and SD of the differences between the two raters were calculated for the upper and lower borders. Intraclass correlation coefficients for the upper and lower borders were calculated using a two-way mixed model for individual ratings (ICC 3,1) (24).

#### Software

SAS Enterprise Guide 8.4 (SAS Institute Inc., Cary, NC, USA) was used to perform statistical analyses and produce figures.

## Results

**Figure 2:**
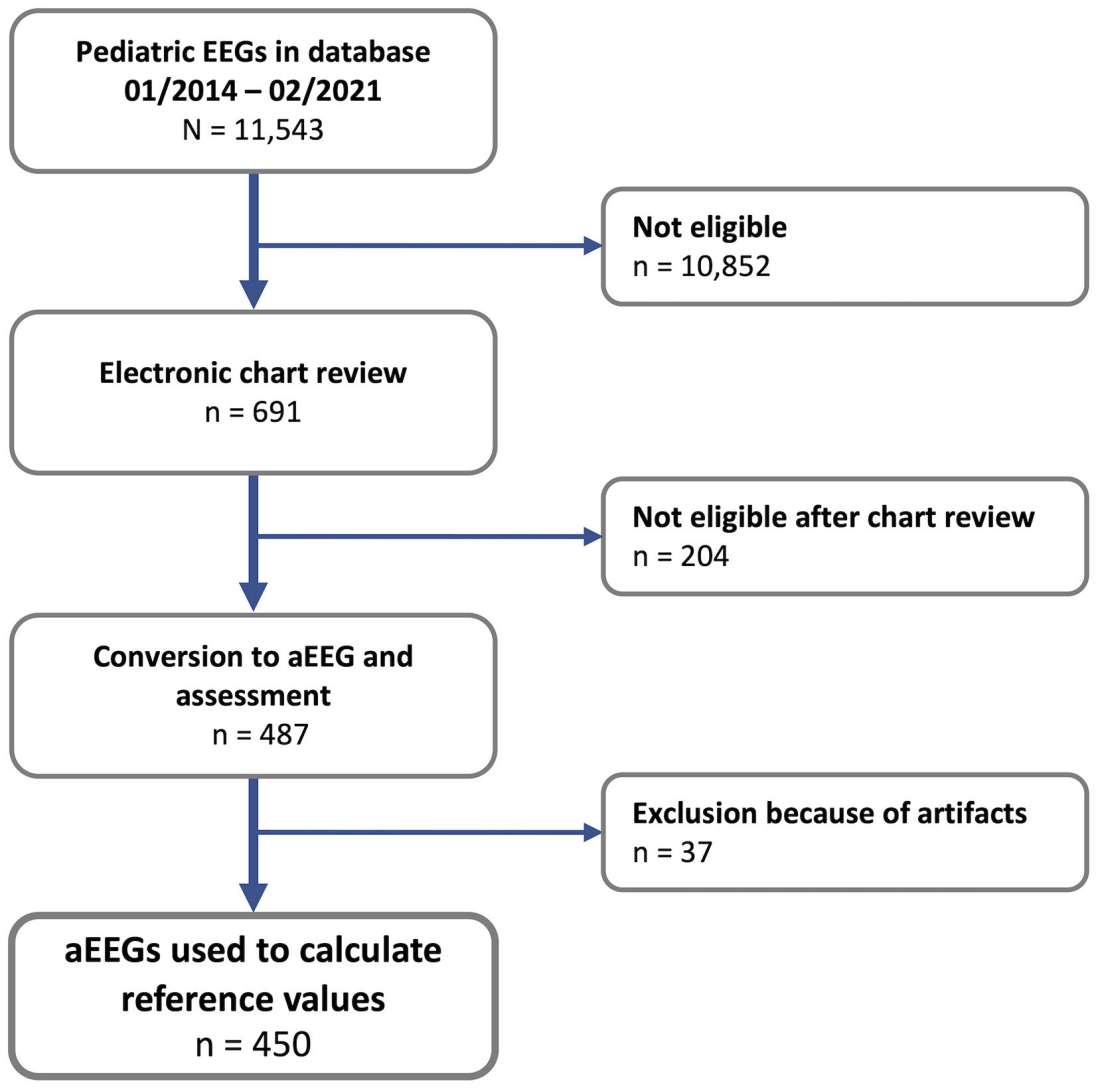
Flow chart of EEG selection and aEEG conversion.

Out of 11,543 EEGs recorded between January 2014 and February 2021 in the Children's Hospital of the University Hospital Essen, 450 unremarkable EEGs were included (Figure 2). The median (IQR) duration was 17 (15–18) min. Males accounted for 52.7 % of the measurements and 60.7 % of EEGs were conducted as routine diagnostics (Table 2). The remaining 39.3 % of EEGs were conducted in suspected neurologic disease, which was ruled out during the diagnostic work-up. None of the patients received a neurologic diagnose later in the history until inclusion into the study.

**Table 2:** Included patients and indications for EEG.

	***n* (%)**	**Mean ±SD**
Male	237 (52.7)	
Female	213 (47.3)	
**Age group**		
<1 year	44 (9.8)	0.5 ± 0.3
1 year	30 (6.7)	1.5 ± 0.3
2–5 years	96 (21.3)	3.6 ± 1.2
6–9 years	79 (17.6)	7.8 ± 1.2
10–13 years	86 (19.1)	12.2 ± 1.2
14–17 years	115 (25.6)	16.1 ± 1.2
**Indication**s		
Routine diagnostics*	273 (60.7)	
Diagnostic work-up in patients with suspected inborn or acquired neurologic disease**	177 (39.3)	

SD, standard deviation; ^*^Before the initiation of therapy or during aftercare, e.g., before or after solid organ transplant, bone marrow transplant, or chemotherapy; ^**^No neurologic disease diagnosed after completion of diagnostics at any timepoint in the patient history (inborn) or before the recording (acquired).

The intraclass correlation coefficient was 0.83 for the upper and 0.87 for the lower amplitude. The mean interrater difference for the upper amplitude was 0.9 μV (SD 6.3 μV) and 0.3 μV (2.2 μV) for the lower amplitude.

**Figure 3:**
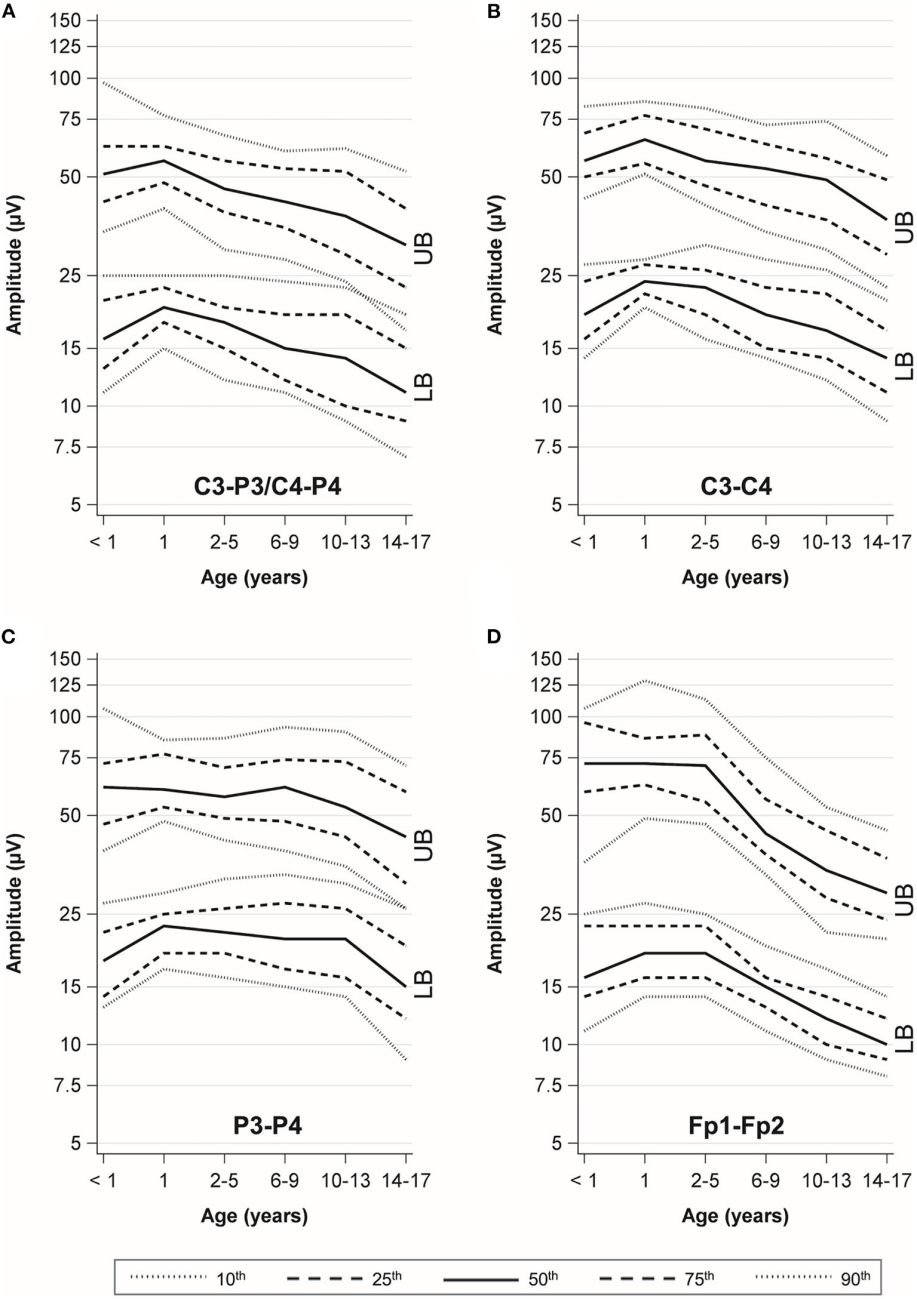
Percentile curves by age. UB, upper border; LB, lower border. **(A)** C3-P3/C4-P4 channel. **(B)** C3-C4 channel. **(C)** P3-P4 channel. **(D)** Fp1-Fp2 channel.

In the C3–P3 and C4 P4 channels, the amplitudes rose from 0 to 1 year of age and showed a continuous decline until the age of 17 years (Figure 3A). A similar trend was observed in all channels, but there were differences in amplitude values and curves between the channels (Table 3, Figures 3B–D). We found statistically non-significant differences in mean amplitudes between males and females (Supplementary Table 1).

**Table 3:** Percentiles by channels and age groups.

**Channel**	**Age**	**Amplitude**	**Percentile [**μ**V]**				
			**10th**	**25th**	**50th**	**75th**	**90th**
C3-P3/ C4-P4	<1 year	Lower	11	13	16	21	25
		Upper	34	42	51	62	97
	1 year	Lower	15	18	20	23	25
		Upper	40	48	56	62	77
	2–5 years	Lower	12	15	18	20	25
		Upper	30	39	46	56	67
	6–9 years	Lower	11	12	15	19	24
		Upper	28	35	42	53	60
	10–13 years	Lower	9	10	14	19	23
		Upper	24	29	38	52	61
	14–17 years	Lower	7	9	11	15	19
		Upper	17	23	31	40	52
C3–C4	<1 year	Lower	14	16	19	24	27
		Upper	43	50	56	68	82
	1 year	Lower	20	22	24	27	28
		Upper	51	55	65	77	85
	2–5 years	Lower	16	19	23	26	31
		Upper	41	47	56	70	81
	6–9 years	Lower	14	15	19	23	28
		Upper	34	41	53	63	72
	10–13 years	Lower	12	14	17	22	26
		Upper	30	37	49	57	74
	14–17 years	Lower	9	11	14	17	21
		Upper	23	29	37	49	58
P3-P4	<1 year	Lower	13	14	18	22	27
		Upper	39	47	61	72	106
	1 year	Lower	17	19	23	25	29
		Upper	48	53	60	77	85
	2–5 years	Lower	16	19	22	26	32
		Upper	42	49	57	70	86
	6–9 years	Lower	15	17	21	27	33
		Upper	39	48	61	74	93
	10–13 years	Lower	14	16	21	26	31
		Upper	35	43	53	73	90
	14–17 years	Lower	9	12	15	20	26
		Upper	26	31	43	59	71
Fp1-Fp2	<1 year	Lower	11	14	16	23	25
		Upper	36	59	72	96	106
	1 year	Lower	14	16	19	23	27
		Upper	49	62	72	86	129
	2–5 years	Lower	14	16	19	23	25
		Upper	47	55	71	88	113
	6–9 years	Lower	11	13	15	16	20
		Upper	33	38	44	56	75
	10–13 years	Lower	9	10	12	14	17
		Upper	22	28	34	45	53
	14–17 years	Lower	8	9	10	12	14
		Upper	21	24	29	37	45

To facilitate bedside interpretation of aEEG, we created a pocket card with a map of the channels according to the 10–20 system, graphical percentiles for each channel, and summary tables with numerical percentile values (Supplementary material).

## Discussion

This is the first study to describe the impact of age and electrode position on aEEG amplitudes and derive age-specific normal values in awake children without underlying or acquired cerebral disease. aEEG amplitudes increased during the 1st years of life, followed by a continuous decline until the age of 17 years. Different amplitude values and curves, but no sex-specific differences were observed between the channels.

The lower margin at all ages and for all channels had a 10th percentile above 5 μV, corresponding to a continuous normal voltage background according to Hellström-Westas et al. (14). This neonatal classification is used up to adult age due to the lack of age-specific reference values (5, 7, 9, 17, 22, 25, 26). According to our data, a normal pediatric aEEG amplitude from any of the analyzed channels fulfills the continuous normal voltage criteria by Hellström-Westas. Therefore, defining “normal” based on this classification in the pediatric aEEG studies mentioned above seems justified. The different amplitudes we found between channels show the impact of electrode positions on the aEEG background. This should be considered when comparing study findings and designing future investigations.

The effect of pathologies and sedatives on the aEEG background remains largely uninvestigated. In conventional EEG, abnormal oscillatory patterns are induced by anesthetic and sedative drugs (27). These patterns reflect a patient's current level of anesthesia, making it possible to track the brain states of a patient (27). Intracranial injuries, a missing skullcap after decompressive craniectomy, modified electrode positions due to head dressings, and external injuries to the head can impact the raw EEG pattern and consecutively the aEEG amplitudes obtained. This calls for caution when applying aEEG normal values in patients with intracranial injury. With respect to background pattern changes over time, the percentiles may help to recognize deterioration or normalization.

The main limitation of our study is the fact that all measurements were performed in awake children. Many children undergoing aEEG monitoring in the PICU receive sedatives, suffer from intracranial pathologies or fall asleep during part of the recording. We previously showed that amplitudes differ between deep and light sleep in healthy children (13, 28), making the establishment of normal values during sleep substantially more difficult. For this reason, we focused on awake aEEGs as a first step toward a systematic aEEG assessment. Intracranial injuries such as large subdural hematoma or trapped air after neurosurgery make the expected aEEG amplitudes unpredictable. This must be considered when using the percentiles for interpretation, as well as the influence of sedatives and other neuroactive medication. In the frontal channels, muscle activity affects the amplitudes (29). This must be considered in patients without muscle activity. aEEG conversion algorithms differ between manufacturers and are not openly accessible (30, 31). Therefore, the same aEEG tracing may have slightly different amplitudes depending on the software used. Further, the manual amplitude measurement may yield different results compared to an automated electronic assessment, limiting the comparability of results between assessment techniques. A disadvantage of automated assessment is the poor performance of software in artifact recognition and removal (32). In contrast, intensive care providers can easily ignore sections containing artifacts during visual bedside assessment.

The aEEG itself has limitations compared to full-channel EEG, as a reduced montage decreases the sensitivity for the detection of seizures (33). Further, aEEG contains no information on waveform morphology and is restricted to a narrow bandwidth with no information on the relative frequency content. For this reason, aEEG should be considered as a complementary or bridging technique until full-channel EEG is available. The findings of this study point out the need to account for age and electrode positions when interpreting aEEGs in infants and children. Further research must determine if amplitude deviations from the here-defined percentiles are associated with pathologies, medication, cerebral dysfunction or outcomes.

With respect to older patients, the results of this study may give some guidance about physiological aEEG amplitudes in young adults, given that there is not a large expanse of differences between baseline EEGs in adolescents versus young adults. However, the ongoing amplitude changes we observed until adolescent age call for systematic investigation of adult baseline aEEG to assess age-dependent physiological variations.

## Conclusion

This study shows that age and electrode position substantially impact aEEG amplitudes. It further provides normal values for bedside assessment derived from awake children without cerebral pathology. The percentiles provided can contribute to the identification of normal and patterns and deviations. Patient age and electrode position, along with medications and pathologies that may affect electrocortical activity, must be considered upon interpretation.

## Data availability statement

The raw data supporting the conclusions of this article will be made available by the authors, without undue reservation.

## Ethics statement

The studies involving human participants were reviewed and approved by Ethics Committee of the Medical Faculty of the University of Duisburg-Essen. Written informed consent from the participants' legal guardian/next of kin was not required to participate in this study in accordance with the national legislation and the institutional requirements.

## Author contributions

NB conceptualized the study, acquired funding, collected and analyzed the data, drafted the initial manuscript, and revised the manuscript. SG and VL collected and interpreted data and participated in the drafting of the initial manuscript. AD interpreted the original EEG. CD-S and UF-M helped to conceptualize analyses and interpret data. All authors critically revised the manuscript for important intellectual content, approved the final manuscript as submitted, and agree to be accountable for all aspects of the work.

## Funding

The study received funding from the Medical Faculty of the University of Duisburg-Essen (Corona Care program) and from the Stiftung Universitätsmedizin Essen. NB received funding from the Medical Faculty of the University of Duisburg-Essen (IFORES program) and from the Stiftung Universitätsmedizin Essen. The funder did not participate in the work.

## Conflict of interest

The authors declare that the research was conducted in the absence of any commercial or financial relationships that could be construed as a potential conflict of interest.

## Publisher's note

All claims expressed in this article are solely those of the authors and do not necessarily represent those of their affiliated organizations, or those of the publisher, the editors and the reviewers. Any product that may be evaluated in this article, or claim that may be made by its manufacturer, is not guaranteed or endorsed by the publisher.
