# Supplementary material for: The impact of age and electrode position on amplitude-integrated EEGs in children from 1 month to 17 years of age
Source: Front Neurol. 2022 Aug 25;13:952193. doi: 10.3389/fneur.2022.952193 (PMC9452771; doi:10.3389/fneur.2022.952193)

Reference values for pediatric aEEG

Channels according to the 10-20 system

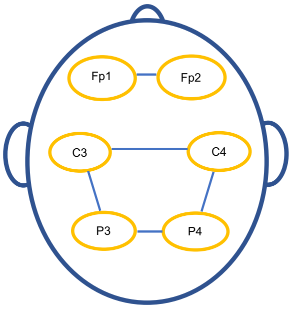

Amplitude estimation

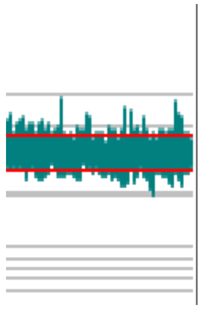

For best comparability, locate the aEEG electrodes according to one or several of the channels above for best comparability

To assess the upper and lower amplitudes, try to estimate the margins of the main aEEGs, ignore solitary spikes and sections with artifacts

These percentiles were deduced from awake healthy children. Use in critically ill, asleep or sedated children requires cautious evaluation of comcomitant circumstances that may alter EEG activity or affect the plausibility of the measurement!

Percentile values

Age	Channel		Percentile 10th	25th	50th	75th	90th
< 1 year	C3-P3/C4-P4	Lower	11	13	**16**	21	25
		Upper	34	42	**51**	62	97
	C3-C4	Lower	14	16	**19**	24	27
		Upper	43	50	**56**	68	82
	P3-P4	Lower	13	14	**18**	22	27
		Upper	39	47	**61**	72	106
	Fp1-Fp2	Lower	11	14	**16**	23	25
		Upper	36	59	**72**	96	106
1 year	C3-P3/C4-P4	Lower	15	18	**20**	23	25
		Upper	40	48	**56**	62	77
	C3-C4	Lower	20	22	**24**	27	28
		Upper	51	55	**65**	77	85
	P3-P4	Lower	17	19	**23**	25	29
		Upper	48	53	**60**	77	85
	Fp1-Fp2	Lower	14	16	**19**	23	27
		Upper	49	62	**72**	86	129
2 - 5 years	C3-P3/C4-P4	Lower	12	15	**18**	20	25
		Upper	30	39	**46**	56	67
	C3-C4	Lower	16	19	**23**	26	31
		Upper	41	47	**56**	70	81
	P3-P4	Lower	16	19	**22**	26	32
		Upper	42	49	**57**	70	86
	Fp1-Fp2	Lower	14	16	**19**	23	25
		Upper	47	55	**71**	88	113
6 - 9 years	C3-P3/C4-P4	Lower	11	12	**15**	19	24
		Upper	28	35	**42**	53	60
	C3-C4	Lower	14	15	**19**	23	28
		Upper	34	41	**53**	63	72
	P3-P4	Lower	15	17	**21**	27	33
		Upper	39	48	**61**	74	93
	Fp1-Fp2	Lower	11	13	**15**	16	20
		Upper	33	38	**44**	56	75
10 - 13 years	C3-P3/C4-P4	Lower	9	10	**14**	19	23
		Upper	24	29	**38**	52	61
	C3-C4	Lower	12	14	**17**	22	26
		Upper	30	37	**49**	57	74
	P3-P4	Lower	14	16	**21**	26	31
		Upper	35	43	**53**	73	90
	Fp1-Fp2	Lower	9	10	**12**	14	17
		Upper	22	28	**34**	45	53
14 - 17 years	C3-P3/C4-P4	Lower	7	9	**11**	15	19
		Upper	17	23	**31**	40	52
	C3-C4	Lower	9	11	**14**	17	21
		Upper	23	29	**37**	49	58
	P3-P4	Lower	9	12	**15**	20	26
		Upper	26	31	**43**	59	71
	Fp1-Fp2	Lower	8	9	**10**	12	14
		Upper	21	24	**29**	37	45

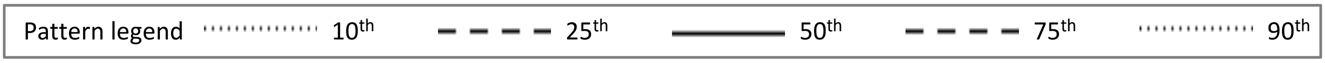

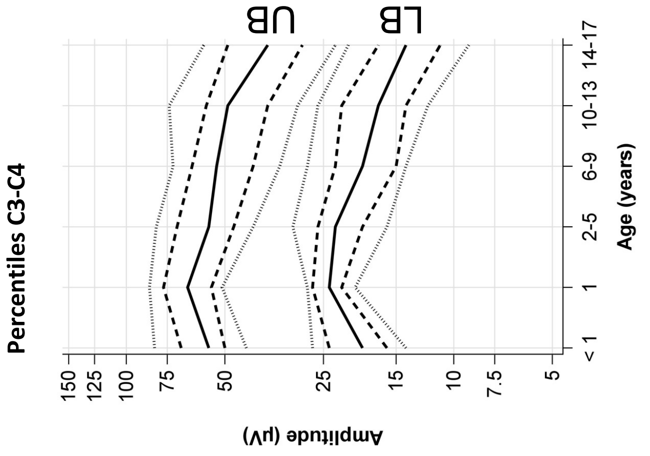

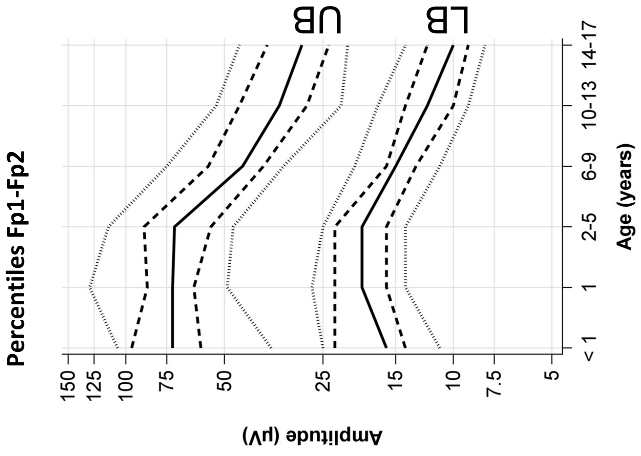

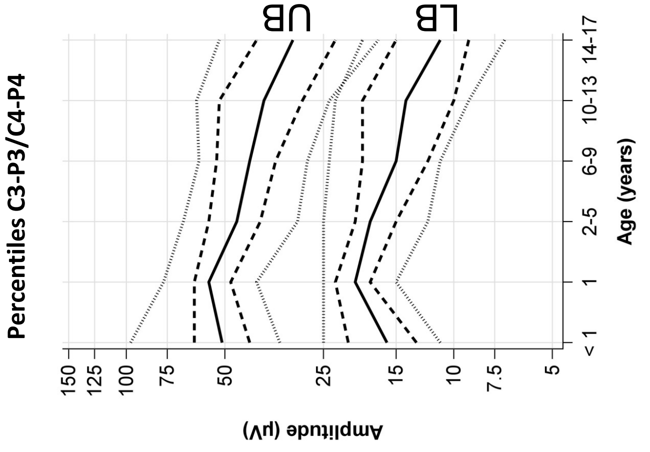

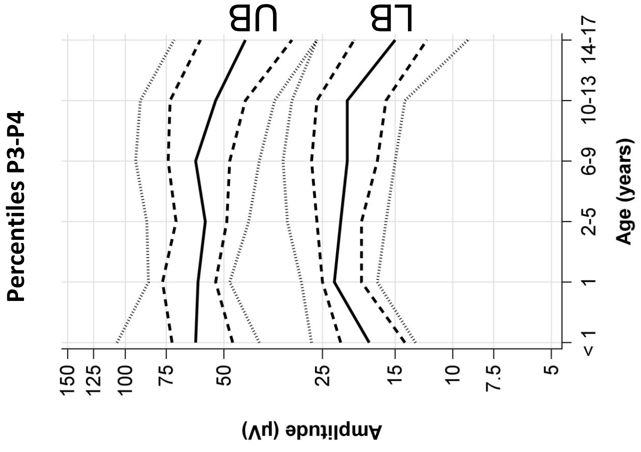

Supplement: Supplementary file 1 [file Data_Sheet_1.PDF]
