# Supplementary material for: The impact of age and electrode position on amplitude-integrated EEGs in children from 1 month to 17 years of age
Source: Front Neurol. 2022 Aug 25;13:952193. doi: 10.3389/fneur.2022.952193 (PMC9452771; doi:10.3389/fneur.2022.952193)
Supplement: Supplementary file 2 [file Table_1.DOCX]

**Supplementary table:** Mean (95 % CI) amplitude values (µV) by sex and age group

|  |  |  | **Channel** |  |  |  |  |  |  |  |
| --- | --- | --- | --- | --- | --- | --- | --- | --- | --- | --- |
|  |  |  | **C – P** |  | **P3 – P4** |  | **C3 – C4** |  | **Fp1 – Fp2** |  |
| **Agegroup** | **Sex** | **n** | **Lower** | **Upper** | **Lower** | **Upper** | **Lower** | **Upper** | **Lower** | **Upper** |
| < 1 year | Male | 24 | 16.0  (14.6 – 17.3) | 54.7  (47.8 – 61.5) | 18.5  (16.3 – 20.7) | 65.4  (54.6 – 76.3) | 18.9  (17.2 – 20.7) | 60.9  (51.4 – 70.4) | 18.0  (15.4 – 20.6) | 82.6  (71.2 – 94.1) |
|  | Female | 20 | 19.2  (16.7 – 21.7) | 60.5  (51.6 – 69.4) | 20.1  (17.0 – 23.2) | 63.1  (51.4 – 74.9) | 20.7  (17.9 – 23.4) | 63.2  (52.8 – 73.5) | 17.2  (14.6 – 19.8) | 66.3  (55.6 – 77.0) |
| 1 year | Male | 21 | 20.2  (19.0 – 21.3) | 55.4  (51.8 – 59.0) | 22.5  (20.5 – 24.4) | 60.8  (55.5 – 66.2) | 24.3  (22.8 – 25.8) | 65.2  (59.5 – 70.8) | 19.3  (17.2 – 21.4) | 82.0  (67.0 - 97.1) |
|  | Female | 9 | 21.2  (18.5 – 23.8) | 60.4  (51.2 – 69.6) | 23.5  (19.3 – 27.6) | 71.4  (56.3 – 86.6) | 24.7  (19.3 – 30.0) | 72.0  (50.9 – 93.0) | 19.8  (16.0 – 23.5) | 73.2  (62.0 – 84.4) |
| 2 – 5 years | Male | 46 | 18.4  (17.3 – 19.6) | 49.1  (45.8 – 52.5) | 22.9  (20.6 – 25.2) | 60.6  (54.2 – 67.0) | 23.7  (21.7 – 25.7) | 61.5  (54.8 – 68.1) | 19.4  (18.0 – 20.9) | 74.4  (67.5 – 81.4) |
|  | Female | 50 | 17.8  (17.0 – 18.7) | 47.0.  (44.7 – 49.3) | 23.1  (21.6 – 24.6) | 60.5  (56.3 – 64.6) | 22.3  (21.0 – 23.5) | 57.8  (54.0 – 61.6) | 19.4  (17.7 – 20.4) | 75.9  (68.1 – 83.7) |
| 6 – 9 years | Male | 49 | 16.7  (15.7 – 17.6) | 45.8  (43.0 – 48.6) | 23.7  (21.9 – 25.6) | 65.7  (60.2 – 71.2) | 20.8  (19.2 – 22.4) | 55.8  (51.6 – 60.1) | 15.4  (14.5 – 16.3) | 50.7  (46.3 – 55.1) |
|  | Female | 30 | 15.3  (14.0 – 16.7) | 41.7  (38.4 – 44.9) | 21.1  (18.6 – 23.7) | 58.7  (51.9 – 65.5) | 18.6  (16.6 – 20.6) | 50.5  (45.0 – 56.1) | 14.1  (12.9 – 15.2) | 44.5  (39.0 – 50.0) |
| 10 – 13 years | Male | 46 | 16.0  (14.5 – 17.5) | 42.0  (38.4 – 45.5) | 23.0  (20.2 – 25.8) | 61.2  (54.0 – 68.3) | 18.8  (17.1 – 20.6) | 50.8  (45.7 – 55.9) | 12.8  (11.8 – 13.7) | 38.8  (34.7 – 43.0) |
|  | Female | 40 | 14.3  (13.1 – 15.4) | 39.1  (36.0 – 42.3) | 21.4  (18.8 – 24.0) | 57.6  (50.4 – 64.7) | 17.6  (15.6 – 19.6) | 48.3  (42.7 – 53.9) | 12.1  (11.2 – 13.0) | 36.3  (31.7 – 41.0) |
| 14 – 17 years | Male | 51 | 12.9  (11.9 – 14.0) | 34.9  (32.2 – 37.6) | 17.4  (15.6 – 19.2) | 49.3  (44.0 – 54.6) | 15.1  (13.7 – 16.5) | 41.5  (37.6 – 45.5) | 10.4  (9.8 – 11.0) | 30.5  (28.2 – 32.7) |
|  | Female | 64 | 11.2  (10.4 – 12.1) | 31.8  (29.5 – 34.0) | 15.6  (14.0 – 17.3) | 43.9  (39.3 – 48.5) | 13.9  (12.6 – 15.2) | 37.9  (34.4 – 41.4) | 10.6  (9.9 – 11.3) | 31.9  (28.7 – 35.2) |
